# Ethnic diversity in UK bioscience: Structural biases and recommendations for action

**DOI:** 10.1371/journal.pone.0339301

**Published:** 2025-12-31

**Authors:** Yusra Siddiqui, Prachi Stafford, Emmanuel Adukwu, Nicholas Freestone, Lovleen Tina Joshi, Emma Yhnell, Katharine Hubbard

**Affiliations:** 1 Department of Biosciences, University of Exeter, Exeter, United Kingdom; 2 Department of Biomedical Sciences, University of Birmingham, Birmingham, United Kingdom; 3 School of Biosciences and Chemistry, Sheffield Hallam University, Sheffield, United Kingdom; 4 School of Applied Sciences, College of Health, Science and Society, UWE Bristol, Frenchay Campus, Bristol, United Kingdom; 5 School of Life Sciences, Pharmacy and Chemistry, Kingston University, Kingston, United Kingdom; 6 Peninsula Dental School, Faculty of Health, University of Plymouth, Plymouth, United Kingdom; 7 School of Biosciences, Cardiff University, Cardiff, United Kingdom; 8 School of Natural Sciences, University of Hull, Hull, United Kingdom; 9 Learning Enhancement and Academic Practice, Buckinghamshire New University, Wycombe, United Kingdom; Instituto Tecnologico Autonomo de Mexico, MEXICO

## Abstract

Scientific research is more innovative and impactful when scientists are drawn from diverse backgrounds. However, the scientific community does not reflect the demographics of wider society. Global Majority communities remain underrepresented within science, particularly within senior leadership roles. Here we explore ethnic diversity within UK academic biosciences, using publicly available staff and student demographic data from the UK Higher Education Statistics Authority. We use the Simpson index as a quantitative measure of diversity within the biosciences and construct linear mixed effect models to identify significant predictors of both student and staff ethnic diversity. For staff, there were significant negative effects on diversity for those in teaching-only roles, and lower diversity amongst higher paid and professorial or senior management staff. For students there are significant negative effects on diversity for part-time students and for postgraduate research students, with PhD students significantly less diverse than undergraduates. There was also considerable variation in diversity by biology subdiscipline, with higher ethnic diversity amongst biomedical sciences and biomolecular subdisciplines, while very low ethnic diversity was found within zoology, plant science and ecology. A correlation was found between the ethnic diversity of bioscience subdisciplines and the diversity of institutions offering those subdisciplines. Based on these findings, we provide evidence-based recommendations for action at various levels, including teaching teams, research groups, bioscience departments and professional bodies. We call upon the sector to take decisive steps to enhance outcomes and experiences for Global Majority staff and students, ultimately diversifying the academic pipeline to the direct benefit of UK bioscience.

## Introduction

Diverse scientific teams have increased innovation, improved problem-solving skills, and stronger research outcomes [1–3]. Studies have demonstrated that diverse teams generate more novel impactful science, are cited more frequently and have stronger interdisciplinary collaborations [4–8]. In the broader economy, companies with equitable and inclusive working environments see better employee engagement, morale and retention [9]. They also create more innovative and creative working environments, resulting in higher profitability and business success [9,10]. Productivity benefits are particularly apparent when there is diversity amongst senior leadership teams [11]. Diversity has also been shown to have a positive impact on group decision making; for example, diverse teams make more reliable and robust jury decisions [12]. Equity, diversity and inclusion is therefore not just a matter of social justice, but has significant tangible benefits and material gain for employers and society [13].

In the UK and elsewhere, the scientific workforce does not reflect the demographics of wider society [14,15]. For example, the UK STEM (Science Technology Engineering and Mathematics) workforce has significantly lower proportions of women, disabled and Black scientists than the UK working-age population [15,16]. Gender and ethnicity imbalances vary significantly by STEM sector; engineering is particularly dominated by White males. While women and Global Majority employees are overrepresented in the health workforce, they are underrepresented within senior and management roles [15]. Specifically within the science and maths workforces, people of Indian ethnicity are overrepresented, but Black, Bangladeshi and Pakistani scientists are underrepresented [15].

This study focuses on ethnic diversity within UK biosciences, exploring publically available demographic data to identify issues at subdiscipline level and to identify potential areas of future focus. Before going further, we clarify our use of language in this space, which is both contested and controversial [17]. We distinguish between ‘race’ and ‘ethnicity’, seeing race as a social construct that has been used to create a hierarchical societal power structure to justify exploitation and oppression, and that has no valid biological basis [18,19]. We use ethnicity to reflect diversity based on cultural identity, customs, and biological ancestry, although note that biological and cultural components of diversity may not correlate. Ethnicity is the term used in the UK HE policy landscape, hence is our choice of language throughout.. We use the language of Global Majority as a more respectful construct to describe communities than the commonly used ‘BAME’ (Black, Asian, Minority Ethnic) [20,21]. Our analysis uses the groupings of ‘White’, ‘Black’ and ‘Asian’ as these are used by public bodies in the UK. However we recognise and acknowledge that these are crude categories that can obscure important differences between groups, and that categorisation itself oversimplifies diversity of populations [18,22].

### The importance of ethnic diversity in the biosciences

The biosciences make essential and varied contributions to major global challenges, including food security, infectious disease monitoring and treatment, loss of biodiversity, and responses to the climate emergency. Global Majority communities disproportionately feel the effects of these crises, further increasing inequity at international scale [23]. These major challenges require collaboration between global communities, scientific researchers, governments, non-governmental organisations and local communities. However, local communities and indigenous populations are often excluded from development programmes [24]. A lack of researcher diversity can also lead to exploitative practices and a disregard for non-Western expertise [18,25,26]. The practice of ‘parachute science’ is a widespread issue, whereby Western researchers conduct research in less wealthy countries without partnering with local experts, or without crediting local experts for their essential contributions [26].

A lack of ethnic diversity in healthcare settings has been linked to worse clinical outcomes for Global Majority patients [27]. For example, outcomes for Black newborns in intensive care contexts are significantly better if treated by a Black clinician [28]. This can be due to lack of training in clinical assessment of Black babies, stereotyping, implicit bias or racism that impact on trust and communication between clinicians and families [29,30]. The proximity of the life sciences to healthcare means that ethnic diversity in the biosciences may affect what research is done, and therefore what treatments might subsequently be developed. For example, the vast majority of participants in Genome Wide Association Studies (GWAS) are of European ancestry, meaning that genetic variation in Global Majority populations is less well understood and opportunities to understand genetic components of disease are lost [31]. A bias towards White patients in training and educational resources means that skin conditions are either missed or misdiagnosed in patients with dark skin [32]. Biomedical diagnostic devices such as oximeters are typically calibrated on lighter-skin with lower melanin levels, leading to overestimation of oxygen saturation and potential missed hypoxemia diagnosis in Black patients [33,34]. If scientists are recruited disproportionately from particular socioeconomic and cultural backgrounds, these biases will persist and ultimately contribute to poorer health outcomes for Global Majority communities.

### Why are STEM disciplines lacking in diversity?

The lack of diversity in the STEM workforce can be attributed to multiple structural issues. These issues start within school level education. Global Majority students often face systemic challenges, such as limited access to high-quality STEM education and extracurricular opportunities in schools, particularly in underfunded areas where minority populations are more concentrated [35]. Additionally, there is a lack of representation and diverse role model visibilty within academia, which can discourage young people from pursuing careers in these fields. Findings from a 14 year UK based longitudinal study reveals that students from the most disadvantaged backgrounds abandon STEM degrees at approximately twice the rate of their peers from more privileged backgrounds [36,37].

Biases in recruitment, hiring, and promotion practices exacerbate these issues. In a Western context, Global Majority individuals have a lower success rate for hiring and promotions, reflecting persistent ethnicity based discrimination [38–40]. Studies where identical fabricated job applications are presented with different gender and ethnicity information (e.g., names, photographs) demonstrate that Global Majority candidates are less likely to be successful even with identical experience [39,40], although some studies do not find significant ethnicity based differences [23]. This effect is also observed in the biosciences; a study focussing on applications for a lab manager role in the United States of America demonstrated an ethnicity bias in favour of Asian candidates, but against Black and Latinx applicants [41]. This bias is intersectional, with multiple studies demonstrating that Global Majority names are more of a disadvantage for male candidates than female [38]. These disparities may represent unconscious or implicit biases, but in other cases may represent direct ethnicity based discrimination [42]. Relative lack of hiring and promotion success for Global Majority individuals creates a vicious cycle whereby leadership teams do not diversify, so are more likely to perpetuate bias and prevent meaningful change [43]. These biases not only limit the career prospects of talented individuals but also deprives the bioscience sector of high-quality research and innovation driven by diverse perspectives.

### Ethnicity within the UK academic context

There is widespread frustration with the ‘remarkably complacent culture’ around racial equality in UK Higher Education [44]. Staff working in UK HE have historically been disproportionately White, although the sector is slowly becoming more ethnically diverse. Between 2003/04 and 2019/20, the proportion of White staff has decreased from 91.4% to 84.6%, while the proportion of Global Majority staff members has nearly doubled from 8.6% to 15.4% [45]. This is broadly comparable to English and Welsh data from the 2021 census, where Global Majority individuals represent 18% of the population. However, Black staff represent only 2.8% of UK staff in HE [45], compared with 3.3% of the total population in the 2021 census [46]. Significant inequity also exists in terms of contract types with lower proportions of Black, Asian and minority ethnic staff on open-ended and permanent contracts, in senior management positions, in professorial roles and on higher salary bands in comparison to White staff [45]. In the UK, 4.3% of all professors are Asian, whereas only 0.7% are Black [45]. This is more extreme in senior positions, with 165 Black professors among a total of 23,515 across the country, and only 2 Black Vice-Chancellors [45,47]. There have been multiple calls to ensure to address the ethnicity pay gap and the lack of ethnicity within academia, particularly at senior leadership level [48–51].

The relative lack of Global Majority individuals within UK academic staff is not reflected in the student population. Global Majority students are overrepresented in undergraduate and postgraduate populations relative to the UK population; 25% of all UK students are from Global Majority communities [52], compared with only 18% of the population [46]. White students have the lowest entry rate to higher education, with 32% of 18 year olds accepted into HE in 2022, compared with 70% of Chinese students and 50% of Black students [53]. However, Global Majority undergraduates are significantly less likely to achieve high outcomes. There is a well documented ethnicity awarding gap within UK HE, whereby Black and Asian students are less likely to obtain a first class or upper second class degree than White peers, even when controlling for grades on entry to university [54–56]. While there are multiple flaws with the UK construction of the awarding gap [22], this relative lack of academic success means that Black and Asian students are less likely to be accepted onto postgraduate programmes with selective entry requirements. This is reflected in a narrowing of ethnic diversity through the academic qualification pipeline; 73% of UK undergraduates are White, rising to 76% of postgraduate taught (MSc/MA) students and 81% of postgraduate research (MRes/PhD) students [52].

### Study aims

This study aims to generate a detailed profile of the ethnic diversity of UK academic staff and students specifically within the biosciences. We use publicly available data from the Higher Education Statistics Authority (HESA) to quantify the relationship between ethnic diversity and a range of relevant characteristics of staff (e.g., salary, professorial status) and students (e.g., undergraduate vs postgraduates, part time vs full time study). We also explore sub-discipline level differences in ethnicity for student populations, and determine whether differences in ethnic diversity within the biosciences are related to institutional diversity. This approach gives us a comprehensive picture of ethnicity representation for the whole of UK academic bioscience, which we then use to make specific recommendations for practice.

## Methods

### Data sources

Staff and student data was downloaded from the HESA website for the years 2019/20, 2020/21 and 2021/22 (Staff DT025 Table 15; Students DT051 Table 45). For all barcharts we present the mean of the three years of data, while modelling includes each year separately (see below). For data protection reasons, HESA round values to the nearest 5; a record of 0 may therefore actually contain 0, 1 or 2 individuals. This gives some small discrepancies in n values presented through the analysis, depending on the rounding level and approach used by HESA to calculate totals. The HESA rounding approach may distort calculated proportions in the smallest cohorts, but rounding to the nearest 5 individuals has minimal impact in most of the population sizes presented here.

Staff datasets include information on subject area, salary, academic role (teaching and/or research) and contract level (academics, full professors without line management responsibility, and those in senior academic management roles, e.g., heads of department, who may or may not also be professors). Student datasets included academic (sub)discipline, mode of study (full-time or part-time) and level of study (Undergraduate = UG, Postgraduate Taught = PGT, Postgraduate Research = PGR). Staff subject areas are defined through a standardised set of administrative ‘cost centres’, which uses broader subject categories than the student data sets so the two are not directly comparable. We took staff data from the Bioscience (code 112) and Anatomy and Physiology (AnatPhys; code 106) cost centres only; in some institutions biomedical staff are included within clinical medicine cost centres but it is not possible to identify these staff within the HESA data specifically.

Institution level diversity data was drawn from HESA Tables DT051 Table 5 (Students) and DT025 Table 2 (Staff). UK universities fall into three main mission groups; the Russell Group, Post-92 institutions and others. The ‘Russell Group’ is a self-selected group of 24 universities whose primary mission is research based, recruiting academic staff on the basis of research from a global talent pool. These institutions also typically have higher undergraduate entry requirements, and are viewed by many as an ‘elite’ group within the sector. ‘Post-92’ institutions are those who gained university status after significant changes to UK HE 1992. Some are new institutions, while others are former polytechnic universities who now have university status. These institutions typically have a lower entry tariff and recruit students from a more local area, typically from less socioeconomically advantaged areas. These institutions are usually more teaching focussed, so any research-active academic staff combine research with a higher teaching load. Post-92 institutions are somewhat analogous to community colleges in the USA in terms of diversity and social mobility, although Post-92 institutions provide and award the same degree titles and overall classifications e.g. BSc, MSc, PhD as Russell Groups. While there are other mission groups within the sector, these are not commonly referred to in UK HE policy, so we group institutions outside both the Russell Group and Post-92s as ‘other’. It should be noted that mission group is not a proxy for quality; many Post-92 institutions outperform Russell Group institutions in university league tables and national quality assessments such as the Teaching Excellence Framework.

Because UK universities do not use a standardised set of academic titles (lecturer, senior lecturer, reader, associate professor, assistant professor etc.) it is not possible to consistently determine the relationship between academic grade and diversity. We therefore used annual staff salary data as a proxy for seniority, although academic salaries do vary between institutions (post-92 universities typically have lower salaries). To account for inflation between years, we established six salary groups (A-F) based on the category data provided (Table 1). There were only 2 individuals in category A and 56 in category B; to generate meaningful conclusions we removed category A and combined categories B and C for analysis.

**Table 1:** Salary groups used for analysis.

Group	2019−20	2020−21	2021−22	Mean Number of staff
A	<£19,202	<£19,612	<£20,092	2
B	≥ £19,202 and <£25,482	≥ £19,612 and <£25,941	≥ £20,092 and <£26,341	56
C	≥ £25,482 and <£34,189	≥ £25,941 and <£34,804	≥ £26,341 and <£35,326	1539
D	≥ £34,189 and <£45,892	≥ £34,804 and <£46,718	≥ £35,326 and <£47,419	5077
E	≥ £45,892 and <£61,618	≥ £46,718 and <£62,727	≥ £47,419 and <£63,668	3718
F	≥ £61,618	≥ £62,727	≥ £63,668	2285
Total				12677

Data includes staff in the Bioscience and Anatomy and Physiology cost centres. n values indicate mean number of staff across the three years of data. Data are annual contracted salaries.

Ethnicity data was available for the categories of White, Asian, Black, Mixed, other and not known; individuals can choose not to declare their ethnicity to HESA. We include individuals who do not declare their ethnicity in our data presentation and analysis for transparency. In the UK context ‘Asian’ refers primarily to those from South Asia (Indian, Pakistani, Bangladeshi) but also includes Chinese and other Asian backgrounds. The HESA data used is for students with a permanent UK address (HESA DT051 Table 45). ‘Asian’ therefore means undergraduates of Asian heritage who have lived and been educated within the UK, and excludes international students from Asia.

### Data analysis and modelling

All data analysis was conducted in RStudio Version 2024.04.2 running R version 4.4.3. For staff data we modelled the effect of salary group (Table 1), academic role (teaching and research, teaching only, research only), level (junior, professor, senior academic), subject area (Bioscience, Anatomy and Physiology) and year (2019/20, 2020/21, 2021/22). For student data we modelled the effects of biology sub-discipline, level of study (UG,PGT, PGR), mode of study (full-time, part-time) and year (2019/20, 2020/21, 2021/22). For both models, to give a single number to encapsulate ethnic diversity we used the Simpson index as a response variable, calculated using the vegan package [57]. The Simpson index is a commonly used measure of relative diversity used in ecological community analysis, which captures both richness (the number of species) and evenness (the relative abundance of species). When one species dominates an ecosystem this results in low evenness, while high species evenness means that the species present are in more balanced proportions. Evenness is the key parameter in this scenario; the number of ethnicity categories in the HESA data is fixed so richness cannot vary, so the relative proportions of each ethnicity group determines the diversity of the population. As such, Simpson is a more appropriate index than the Shannon index, which gives more weight to rare species, i.e., richness [58]. Simpson is also more appropriate here than alternatives such as the Gini index of inequality which measures evenness of resource distribution not population structure [59]. We acknowledge that the population structure captured by the HESA data has a small number of categories, i.e., richness is low, meaning the Simpson index may saturate where one group dominates [58].

There are multiple indices of diversity, so we selected the Simpson index as it places more emphasis on evenness between species than other diversity measures (e.g., Shannon index), making it more appropriate for the HESA data which has a fixed number of categories [60].

Subdisciplines, mode of study and level of study were independent of one another, but there was pseudoreplication by year due to students on multi-year programmes and staff employed across multiple years. We therefore constructed linear mixed effect models with the lmer package, with year as a random effect and other factors as fixed effects (Table 2). Assumptions of residual normality and homoscedasticity for each model were confirmed by visual inspection of residual plots via the DHARMa package. Three linear models were constructed for each demographic, then optimal model structure determined via Akaike Information Criterion (AIC) comparisons (Table 2). For both staff and students, we fitted a linear mixed model to predict ethnic diversity (Simpson index) with the available explanatory variables (Table 2). P-values for individual model effects were determined via Satterthwaite approximation using the lmerTest package.

**Table 2:** Model structures used during optimisation.

Data	Model	Structure	AIC	Optimum
Staff	1	simpson ~ salary.gp + role + level + subject + (1 | year)	−47.42	x
	2	simpson ~ salary.gp * (role + level) + subject + (1 | year)	−45.49	
	3	simpson ~ role * (salary.gp + level) + subject + (1 | year)	−35.28	
Students	1	simpson ~ subject + mode + level + (1 | year)	−241.59	x
	2	simpson ~ subject * (mode + level) + (1 | year)	−156.14	
	3	simpson ~ (subject + mode) * level + (1 | year)	−179.54	

* Indicates interactions with all terms in brackets. Optimal models for each category are those with the lowest AIC value indicated as ‘x’. Simpson = simpson index of diversity.

## Results

**Fig 1:**
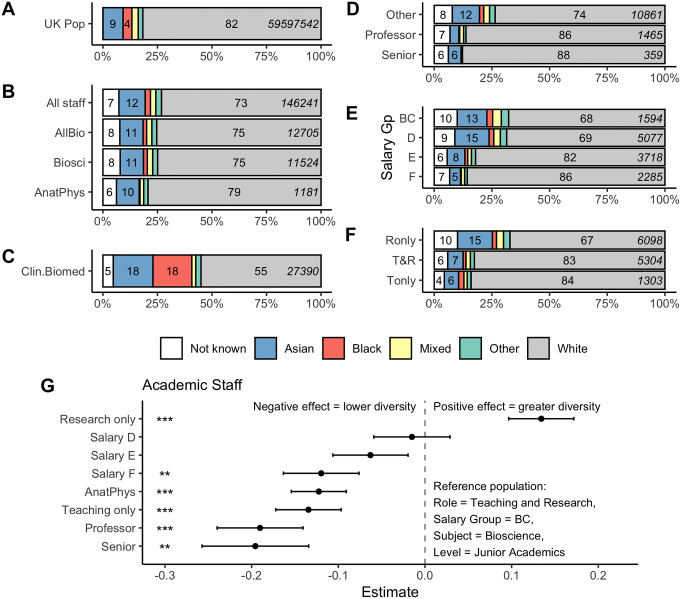
Ethnic diversity of UK academic staff within the biosciences. A: UK population data from the 2021 census for England and Wales. B: Discipline level data comparing all UK academic staff, all staff within the biosciences (AllBio) and staff specifically in the Bioscience (Biosci) or Anatomy and Physiology (AnatPhys) categories C: Ethnic diversity of UK Clinical Biomedical staff. D-F: Ethnic diversity of UK bioscience academic staff by D: professorial status, E: salary group and F: research and/or teaching status (Ronly = Research only, Tonly = Teaching only, T&R = Teaching and Research). For A-F bars are labelled if the relevant category represents 4% or more of the total population indicated in italics. G: Results of linear models. Positive estimates indicate higher Simpson index of diversity, negative estimates lower diversity. *** indicates p < 0.001, ** indicates p < 0.01. Data source HESA Staff Table 15.

We first explored ethnic diversity within UK Bioscience staff (Fig 1). Ethnic diversity among academic staff in the HESA dataset was comparable to that of the UK population (Fig 1A). Of bioscience academic staff, 10.7% are Asian and 1.7% are Black, compared with 9% and 4% respectively in the UK population (Fig 1B). We also obtained ethnicity data for clinical biomedical scientists as an alternate bioscience career destination with publicly available data. The biomedical science population was considerably more diverse than any of the academic staff groups and the UK population (Fig 1C); 18% of clinical staff were Asian and 18% were Black. The overall HESA staff data masks significant differences in ethnic diversity by academic seniority, salary and academic role (Fig 1D-1F). 86.4% of senior academics declare their ethnicity as White. There were 0 bioscience academics identifying as Black within the senior leadership academic category, although this could represent fewer than 5 individuals rounded to 0 by HESA (Fig 1D).

To quantify these effects, we constructed a linear mixed effect model to predict the effects of salary group, academic level and academic role on academic staff ethnic diversity (Simpson index). Within this model, there was a significant negative effect (i.e., lower ethnic diversity) of professors and senior academics (Professors β = −0.19, p < 0.01; Senior β = −0.2, p < 0.01). Academics in the highest paid salary groups were significantly less ethnically diverse than those in the lowest group (Salary Group F β = −0.12, p = 0.01; Fig 1E,1G). There was also a significant effect of academic role; research only staff had a positive effect compared to teaching and research staff (β = 0.13, p < 0.01), while teaching only staff were significantly less ethnically diverse than teaching and research colleagues (β = −0.13, p < 0.01; Fig 1F,1G).

**Fig 2:**
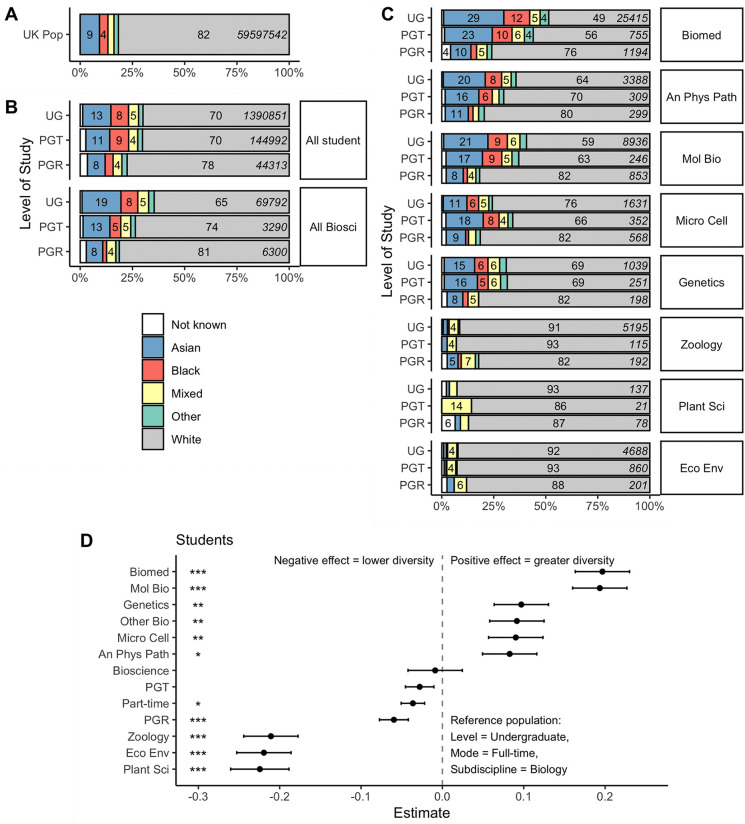
Bioscience student ethnicity. A: Ethnicity of UK Population in 2021 Census. B: Ethnicity of all students and bioscience students by level of study (UG = undergraduate, PGT = postgraduate taught, PGR = postgraduate research) C: Ethnicity by biology subdiscipline and level of study. Bars labelled where group represents at least 4% of total population. Mean number of individuals given in italics on right hand side; data averaged for years 2019/20, 2020/21 and 2021/22. D: Results of linear models giving predictors of Simpson index of diversity; * indicates p < 0.05, ** p < 0.01, *** p < 0.001. Data source: HESA DT051 Table 45.

We conducted the equivalent analysis for undergraduate (UG), postgraduate taught (PGT) and postgraduate research students (PGR) in the Biosciences. 18.6% of undergraduate bioscientists were Asian and 8.1% were Black, indicating bioscience attracts a significant number of Global Majority undergraduates compared with other academic disciplines (Fig 2B). However the proportion of Global Majority students decreased for both postgraduate taught (12.8% Asian, 5.2% Black) and postgraduate research bioscience students (7.9% Asian, 1.9% Black). Compared to the UK population, Black students were therefore overrepresented in the bioscience UG population, but significantly underrepresented in the PGR pool. Asian students were significantly overrepresented in the UG and PGT pools, but the PGR pool was comparable to the UK population in terms of Asian students (Fig 2B). For both groups, there was therefore a loss of Global Majority representation through the academic qualification pipeline.

Within the biosciences subdisciplines there was considerable variation in student ethnic diversity (Fig 2D). Biomedical science undergraduates had the highest ethnic diversity (28.9% Asian, 12.5% Black). Zoology, Plant Science and Ecological disciplines had the lowest proportions of Black and Asian undergraduates (Zoology 2% Asian, 0.8% Black; Ecology and Environmental Biology 1.4% Asian, 0.6% Black; Plant Science 1.5% Asian, 0% Black). There were no Black plant science students in the dataset at any level of study [61], and no Black PGR students in Ecology and Environmental Biology.

We again constructed a linear model to predict ethnic diversity (Simpson index) with subject, mode and level as fixed effects and year as a random effect (Table 2). The model’s total explanatory power is substantial (conditional R^2^ = 0.70) and the part related to the fixed effects alone (marginal R^2^) is of 0.69. Within this model we identified significant positive effects (higher ethnic diversity) for Molecular Biology (β = 0.19, p < 0.01), Biomedical Science (β = 0.2, p < 0.01), Anatomy, Physiology and Pathology (β = 0.08, p = 0.01) and Genetics (β = 0.1, p < 0.01). There were significant negative effects (lower ethnic diversity) for Zoology (β = −0.21, p < 0.01), Ecology and Environmental Biology (β = −0.22, p < 0.01) and Plant Science (β = −0.22, p < 0.01). Studying part-time also had a significant negative effect on ethnic diversity (β = −0.04, p = 0.01).

**Fig 3:**
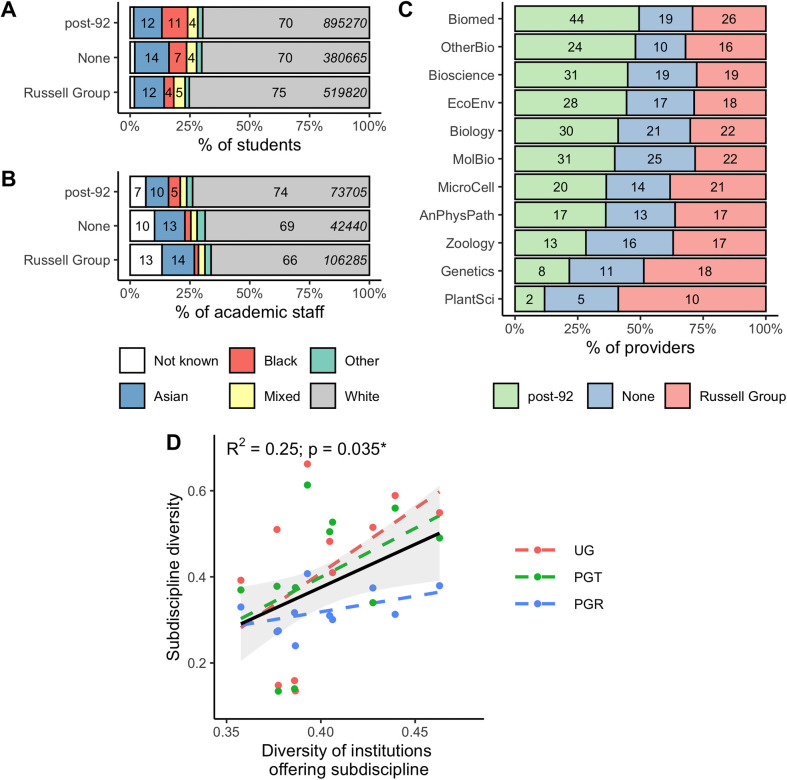
Bioscience ethnic diversity partially reflects institutional diversity. A: Ethnic diversity for all full time students by mission group [i.e., all disciplines not just bioscience]. B: Ethnic diversity for all academic staff by mission group [i.e., all disciplines not just bioscience]. C: Provision of bioscience subdisciplines by institutional mission group. D: Relationship between institutional student diversity for institutions offering each subdiscipline and bioscience subdiscipline diversity. Annotations give results of linear modelling across all three student populations. Data for full-time students only.

We reasoned that institutional type might be a contributory factor to the differences in student ethnic diversity at subdiscipline level. Not all institutions offer all bioscience subdisciplines, and UK HEIs vary considerably in the ethnic makeup of both students and staff. For students, there is lower student ethnic diversity in ‘elite’ research-focussed Russell Group universities than in teaching-focussed post-92 institutions or those with no mission group (Fig 3A). In post-92 institutions 11% of students are Black, compared to only 4% in Russell Groups. For academic staff, post-92 institutions had higher proportions of staff who declared their ethnicity than other institution types (Fig 3B). As a proportion of all academic staff, Russell Group institutions had higher proportions of Asian academic staff (14%) than other institution types, but a lower proportion of Black staff (2%; Fig 3B).

We identified there are differences in the bioscience specialisms offered by post-92, Russell Group and no mission group institutions (Fig 3C). For example, only a small proportion of biomedical sciences programmes are based in ‘elite’ research-focussed Russell Group institutions (26/89 = 29%), while the majority of plant sciences (10/17 = 58%) and many genetics (18/37 = 48%) programmes are offered by Russell Group institutions (Fig 3C). Ethnicity data is not available at the level of individual programmes within the HESA data, so we were unable to distinguish between the contribution of the institution or the subdiscipline to diversity directly (e.g., within a given institution, are biomedical sciences students more ethnically diverse than zoology students). We therefore identified which institutions offered the various subdisciplines and calculated a Simpson’s diversity index for that sub-population of institutions (Fig 3D). We fitted a linear model to predict bioscience subdiscipline diversity (Simpson index) with level of study (UG, PGT, PGR) and institutional diversity as predictors. The model explains a statistically significant and moderate proportion of variance (R^2^ = 0.25, F_(3, 29)_ = 3.29, p = 0.035). The effect of institutional diversity is statistically significant and positive (β = 2.00, t_(29)_ = 2.56, p = 0.016), but level of study did not have a significant effect (PGT p = 0.848; PGR p = 0.107).

## Discussion

This study identifies significant variability in ethnic diversity within UK Biosciences, with substantial losses of Global Majority talent through the academic pipeline. Bioscience postgraduate research populations are significantly less ethnically diverse than undergraduate or postgraduate taught populations. There is also a loss of Global Majority representation through the academic career pipeline, with professors and the highest paid academics having the lowest ethnic diversity. Ethnic diversity is higher within the research staff population, and significantly lower for teaching focussed academics. Within the student population there is considerable variation in the ethnic diversity between bioscience subdisciplines, with biomedical science having the highest diversity and ecology, zoology and plant sciences having significantly lower ethnic diversity than other areas of the biosciences. We also identify that institutional variation in bioscience provision is correlated with ethnic diversity. Those disciplines primarily offered in ‘elite’ research focussed (Russell Group) institutions have lower ethnic diversity (e.g., plant science), and there is a significant relationship between the ethnic diversity of students in a subdiscipline and the ethnic diversity of students in those institutions offering that subdiscipline.

While some of our findings mirror known patterns of decreasing ethnic diversity through the academic pipeline [14,15], focussing on the biosciences specifically gives some novel insights into Equity, Diversity and Inclusion (EDI) issues within STEM. In particular, our analysis identifies that within UK bioscience, many subdisciplines have significantly higher ethnic diversity than the total student population or the national population. This demonstrates that there is considerable appeal of STEM disciplines to Global Majority students. However, our analysis demonstrates that this interest is focussed on particular subject areas, particularly those affiliated with healthcare [62]. Global Majority students may have higher affinity with ‘from STEM’ subjects such as medicine and engineering than ‘in STEM’ subjects such as biology or physics [63]. Our analysis also identifies lower diversity in UK ecological disciplines, compared with molecular biosciences. This lack of diversity in senior ecologists results in fewer same-race role models for students, resulting in a lower sense of belonging and inclusion for Global Majority students in these disciplines [64]. This reduces student interest in staying in ecology, creating a cycle of underrepresentation [64].

We note that our study uses publicly available data on ethnicity, which cannot account for the qualitative factors that generate the inequalities here. Without further research we cannot determine to what extent these issues reflect cultural aspects (e.g., customs, beliefs, values), implicit bias or racial discrimination. Long term longitudinal studies in the UK have identified that there is no difference in intrinsic interest in science at school on the basis of ethnicity [36,37]. However, Global Majority students are less attracted to STEM qualifications and careers for a variety of reasons including dominant cultural representations of scientists as White men, educational gatekeeping, poor career advice, inequity of science capital and the relative appeal of other career options. All of these are shaped by gender, socioeconomic factors and ethnic and cultural factors [37,62,63]. Some of these factors have influence before university. Within those UK students doing A-levels (the post-compulsory academic qualification taken by 17–18 year olds prior to university), biology is taken by 29% of Indian pupils compared with only 15% of White British and 10% of Black Caribbean pupils. The relative visibility of careers related to the sub disciplines may be significant, as subject interest and career aspirations are linked [65]. Within the UK, graduates from medical and medicine-related programmes (including biomedical science) have higher rates of full-time employment than from bioscience and sport science programmes [66], which may influence subject choice. Our study indicates that the biosciences may provide a useful setting within which to explore the relative contributions of these factors further, as we identify significant differences in student ethnic diversity between closely related fields.

Our data highlight that even within those disciplines that attract significant numbers of Global Majority students at undergraduate level, there is a disproportionate loss of these individuals on entry to postgraduate research. This mirrors data from the USA, where Black students in biological sciences have lower enrollment rates into postgraduate programmes than undergraduate, and even lower proportions of students being awarded doctorates [67]. In the UK, Global Majority bioscientists are very well represented within clinical biomedical science roles, so our findings demonstrate it is academic careers specifically that are either unattractive or exclusionary to Black and Asian scientists. It should also be highlighted that most clinical biomedical staff are in roles paid significantly less than their academic counterparts. While clinical bioscientists are essential and lead to rewarding careers, UK bioscience should be concerned that Black bioscientists in particular are disproportionately in lower paid clinical roles and are not well represented in academic research positions. Within academia, it is also of significant concern that Global Majority bioscientists are disproportionately in junior academic roles at lower pay grades.

The significant loss of Global Majority bioscientists through the qualification and career pipeline indicates that there are significant barriers to academic progression. The Black and Asian awarding gap [55] is likely to be highly significant, as obtaining a 1st class/upper second class degree is typically a requirement for admission to PhD programmes. The reasons underpinning the awarding gap are complex and multifaceted [68]. Given the high proportion of Global Majority students in biomedical and molecular biology programmes, it is especially important that curricula in these areas are culturally sensitive and give representation to Global Majority individuals and examples [18,69,70]. There is also a need for appropriate pastoral care, mentoring, sponsorship, careers guidance and support for Global Majority students, particularly for those of Black heritage who are most significantly underrepresented in the research population. Our analysis also identifies that Black and Asian scientists are poorly represented in the teaching focussed academic pool compared with the teaching and research or research focussed populations. This means that undergraduate students in particular may not encounter Global Majority scientists, even when those potential role models may work in the same building but in research spaces rather than teaching spaces.

Our analysis also identifies a previously under researched effect of the relationship between institutional educational provision and ethnic diversity. Our analysis demonstrates that ~25% of the variation in bioscience sub-discipline ethnicity can be attributed to the diversity of the institutions offering that sub-discipline. The resolution of the publicly available datasets used here means that we are triangulating sector level bioscience data with institutional diversity data; we unfortunately do not have bioscience specific ethnicity data at the level of institution. However, our analysis indicates the impact of institution type on bioscience diversity is significant. UK institutions have autonomy over which programmes they offer, meaning that there is uneven distribution of degree programme availability. This has led to some programmes (e.g., plant science, genetics) offered primarily in ‘elite’ Russell Group institutions, while other disciplines (e.g., biomedical sciences) are offered more by Post-92 institutions with greater student diversity.

This institution level effect creates cumulative disadvantage. Global Majority students are less likely to be admitted to Russell Group institutions [71], more likely to be disadvantaged by the awarding gap while studying in Post-92 institutions [55], and less likely to be admitted to postgraduate programmes having not studied at a ‘prestigious’ Russell Group undergraduate institution. However, for UK bioscience to diversify there also needs to be recognition that Global Majority staff and students are disproportionately found in non-Russell Group institutions [72], so diversity initiatives for postgraduate recruitment need to extend to all institution types, not just ‘elite’ institutions.

## Conclusions and recommendations for action

Our data highlight a range of issues relating to Global Majority staff and student representation and progression within UK bioscience. To make meaningful change, everyone within the biosciences needs to recognise and understand these issues and to take meaningful action. Here we make some specific recommendations pertinent to individuals, departments, communities and professional bodies, linked to evidence based practice or existing examples within the biosciences where possible (Table 3).

**Table 3:** Recommendations for action to address issues relating to ethnic diversity in UK biosciences.

Ease of Implementation and impact	Target Area	Action and References	Enablers and Barriers
**Easy but effects felt locally or at the level of individuals**	Representation within resources	Use diverse role models in teaching and research, outreach and recruitment materials [73–75].	An “entry-level” activity that all colleagues should be doing. Many repositories of examples exist (e.g., Scientist Spotlight Repository)
	Building networks	Connect students with Global Majority bioscientists through formal or informal networks (e.g., Black in Plant Science) [61,76–78].	Straightforward assuming that staff have access to diverse networks. Financial and organisational support for formal networks can be provided by institutions and professional bodies.
	Support & Mentorship	Provide tailored mentorship, especially for Black students.	Such schemes may reside at the level of the Department, university or professional body.
	Recognising the impact of racism and discrimination	Recognise that racism exists. Acknowledge the burden that discrimination and EDI work has on minoritised individuals. Provide or signpost to appropriate support [79].	Requires individuals to change mindsets and recognise this burden. May require department level support, including active efforts to cultivate allyship amongst wider colleagues.
**Medium**	Anti-racist Culture	Foster inclusive, anti-racist environments and openly acknowledge systemic barriers. Provide anti-racism training for all staff [25,80–82].	Can be developed by teaching teams but may need central university support and expertise. Individuals need time and support to develop knowledge in these areas. Also needed by professional bodies
	Curriculum Reform	Decolonise curricula, integrate diverse perspectives, and discuss historical inequalities [69,70,73,83–85].	Medium: Individuals may take action, but will have more impact at department level, particularly if supported by learned societies and communities of practice.
	Equity Monitoring	Track ethnic diversity in hiring and advancement; ensure diverse selection panels.	Typically done centrally by HR departments, but departments can take ownership of acting on the data.
	Research Ethics	Foster collaboration and ethical research in global contexts [8,86].	Often driven by funding bodies’ policies, implemented through internal ethics processes
	National Bioscience Policy & Curriculum	Address diversity gaps in biosciences and assess impact; Embed equity and anti-racism in accreditation [87].	Medium: Requires coordinated effort by professional bodies, including through accreditation criteria and mechanisms to improve representation in Prize and Grant awarding.
	Leadership & Recognition by Professional Bodies	Ensure diversity in leadership, awards, and decision-making roles [88].	Medium: Requires ownership and oversight.
	Financial Support for Publishing	Reducing financial burdens/barriers in publishing for underrepresented bioscientists [72].	Medium: Requires prioritisation of funding from journals (e.g., through fee waivers) or support from funding bodies.
**Challenging but with widespread impact**	University Admissions	Use contextualised admissions to support Global Majority talent [89].	University-level oversight needed here with possible changes necessary to policies and procedures.
	Career Progression	Ensure equitable progression and promotion and retention of minoritised staff.	Requires institutional efforts including university-wide mentoring programmes, revisiting promotion criteria, systematic monitoring of data and commitment to change.
	Discipline-Specific Action	Address low ethnic diversity zoology, plant science and ecology-related fields through targeted initiatives [25,61,64].	Challenging: Action required at the level of learned societies and PRSB’s. Often these groupings can be affected by a small number of focussed individuals determined to change national policies.
	Publishing Equity	Promote diverse editorial boards, inclusive writing, and equity in publishing [90,91].	Requires focussed action from the publishing industry

Addressing the ethnic disparities highlighted in this study requires a multifaceted approach, and coordinated actions between multiple stakeholders. Without such interventions, the UK bioscience sector risks continuing to lose valuable talent, thereby limiting potential for scientific progress and innovation.
